# Ectopic opening of the common bile duct and duodenal stenosis: an overlooked association

**DOI:** 10.1186/1471-230X-10-142

**Published:** 2010-12-04

**Authors:** Erkan Parlak, Selçuk Dişibeyaz, Cem Cengiz, Bahattin Çiçek, Yasemin Özin, Sabite Kacar, Nurgül Şaşmaz, Burhan Şahin

**Affiliations:** 1 Türkiye Yüksek İhtisas Hospital, Department of Gastroenterology, Ankara, Turkey

## Abstract

**Background:**

Ectopic opening of the common bile duct into the duodenal bulb (EO-CBD-DB) is a rare disease that may be complicated by duodenal ulcer, deformity, stenosis and biliary stones. The aim of this study is to report clinical presentations, endoscopic diagnosis and treatment of this entity as well as to investigate its association with duodenal stenosis.

**Methods:**

Gastroduodenoscopic findings and radiological imaging were evaluated for ectopic papilla and duodenal stenosis. Diagnostic methods, endoscopic procedures and long-term outcomes of the endoscopic treatment were presented.

**Results:**

EO-CBD-DB was found in 74 (77.1%) of the 96 patients with duodenal deformity/stenosis (79 male, 17 female, mean age: 58.5, range: 30-87 years). The papilla with normal appearance was retracted to the bulb in 11 while it was at its usual location in the remaining 11. The history of biliodigestive surgery was more common in patients with EO-CBD-DB who were frequently presented with the common bile duct stone-related symptoms than the other patients. Thirteen (17.6%) of the patients with EO-CBD-DB were referred to surgery. Endoscopic treatment was completed in 60 (81.1%) patients after an average of 1.7 (range: 1-6) procedures. These patients were on follow-up for 24.8 (range: 2-46) months. Endoscopic intervention was required in 12 (20%) of them because of recurrent biliary problems. Treatment of the patient who had stricture due to biliary injury during laparoscopic cholecystectomy is still continued.

**Conclusions:**

The presence of EO-CBD-DB should be considered particularly in middle-aged male patients who have duodenal deformity/stenosis. Endoscopic treatment is feasible in these patients. The long-term outcomes of endoscopic therapy need to be compared with surgical treatment.

## Background

Ectopic opening of the common bile duct into the duodenal bulb (EO-CBD-DB) is a rare entity that was defined long ago. Its clinical significance as well as endoscopic findings and therapies have been reported as few cases and case series [1-13]. The case series show that the majority of patients with EO-CBD-DB are male, some of them developing duodenal ulcer leading to duodenal deformity (DD) and even stenosis at the duodenal apex (apical stenosis; AS) [7-9]. Interestingly, most of the cases were published from the far east countries and Turkey. This may be a cause of why this entity is not well-recognized in the western countries. Another reason may be that some endoscopists might think these patients have spontaneous biliary fistula. In a group of patients with DD/AS, failure of the procedure because the second part could not be reached or papilla could not be found may also explain why this abnormality is not identified widely. Biliary stone and duodenal ulcer are common complications related with this entity leading to frequent operations especially when EO-CBD-DB could not be identified. In this sudy, EO-CBD-DB was demonstrated in a vast majority of the patients with DD/AS. We herein emphasize their association and important features of endoscopic therapy with its long-term efficacy.

## Methods

The files of the patients who underwent ERCP between January 2005 and August 2008 were reviewed. The data were collected prospectively and analysed retrospectively. The demographic data, presentations, surgical histories, endoscopic procedures, complications and success rates as well as the need for repeat endoscopic procedures on follow-up were determined.

**Figure 1:**
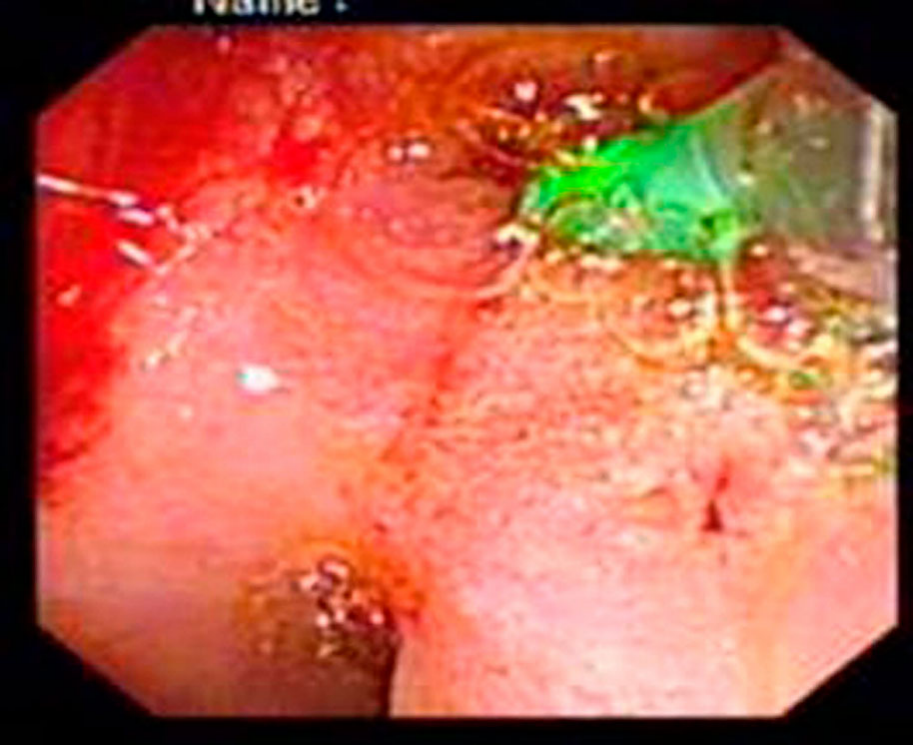
**Typical endoscopic view**. Slit-like biliary and pancreatic orifices easy to cannulate.

**Figure 2:**
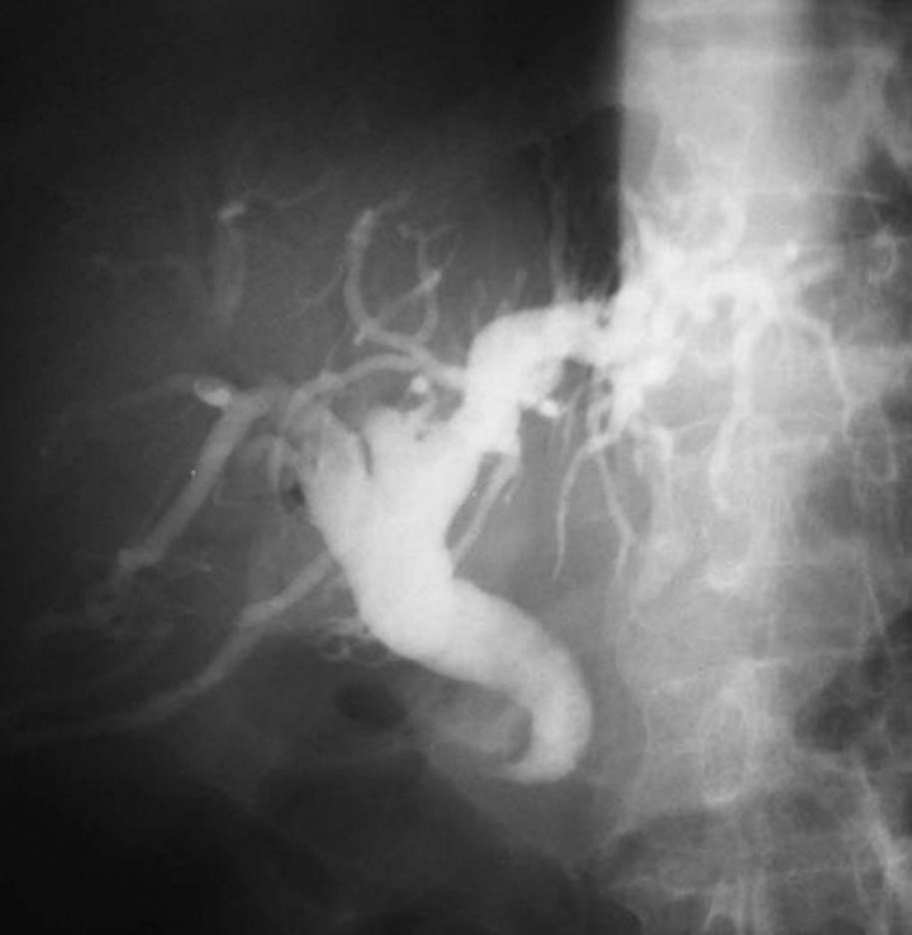
**Typical cholangiogram**. Hook-shaped appearance.

**Figure 3:**
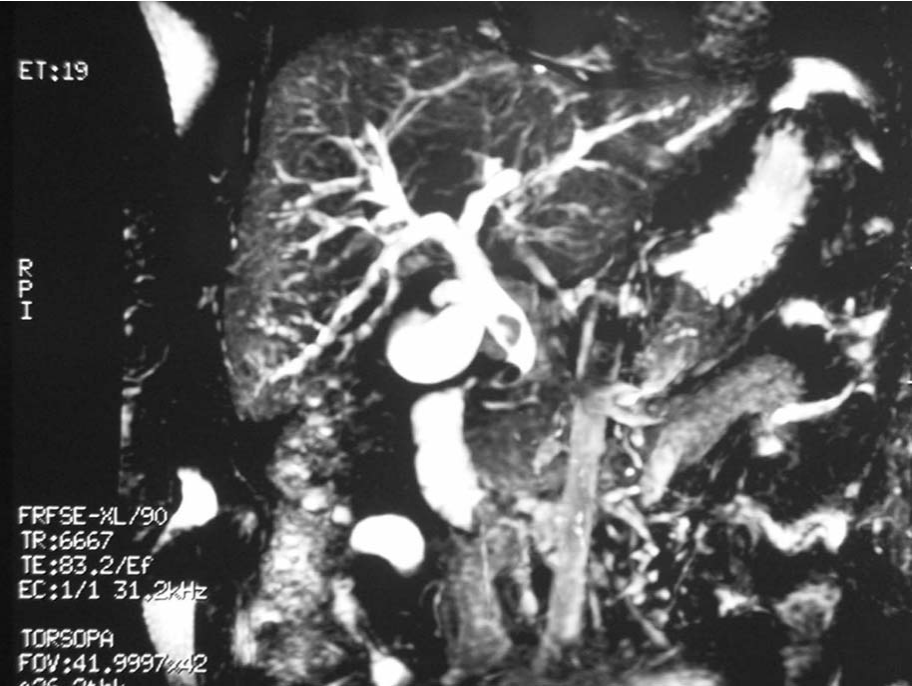
**Magnetic resonance imaging**. Hook-shaped appearance and CBD stones are observed.

The EO-CBD-DB was defined as the failure to demonstrate a papilla in its original location in the second part of the duodenum and observing an opening in the bulb shown to be of CBD on cholangiogram. While duodenoscope is pulled back to the bulb on short position and the right side of the duodenal wall is carefully inspected, the fist papillary structure and orifice in view is the main pancreatic orifice (location where the minor papilla opens could not be identified so far, likely due to duodenal deformity). The orifice of the CBD is a slit-like opening 2-3 mm. above the main pancreatic orifice. Cannulation of CBD is easy to perform and cholangiogram shows typical hook-shaped appearance (Figure 1, 2). In patients with apical duodenal stenosis that did not allow to pass with duodenoscope, duodenoscope is replaced with a gastroscope. Then, an orifice is observed in the bulb by gastroscope and no evidence of a papilla-like structure in the second or third portion can be seen. Cholangiogram is obtained by magnetic resonance cholangio pancreatography (MRCP) or percutaneous transhepatic cholangiography (PTC) in these patients showing typical hook-shaped appearance (Figure 3).

**Figure 4:**
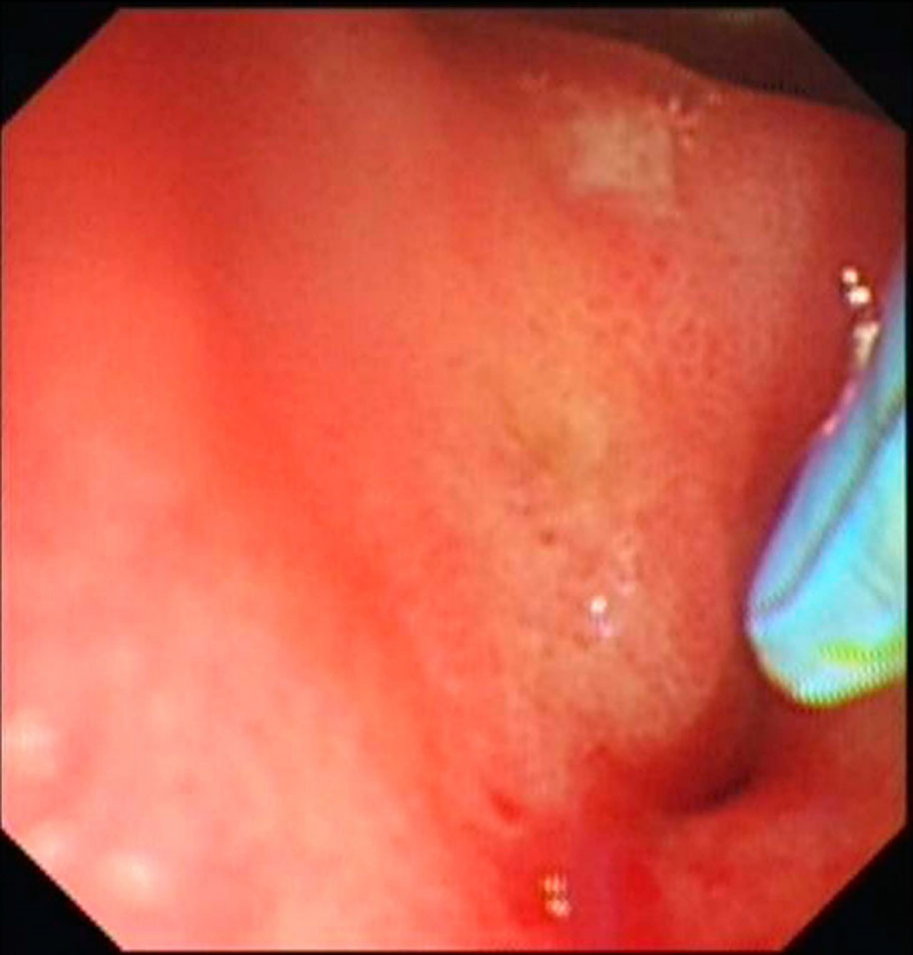
**CBD orifice proximal to apical stenosis**.

**Figure 5:**
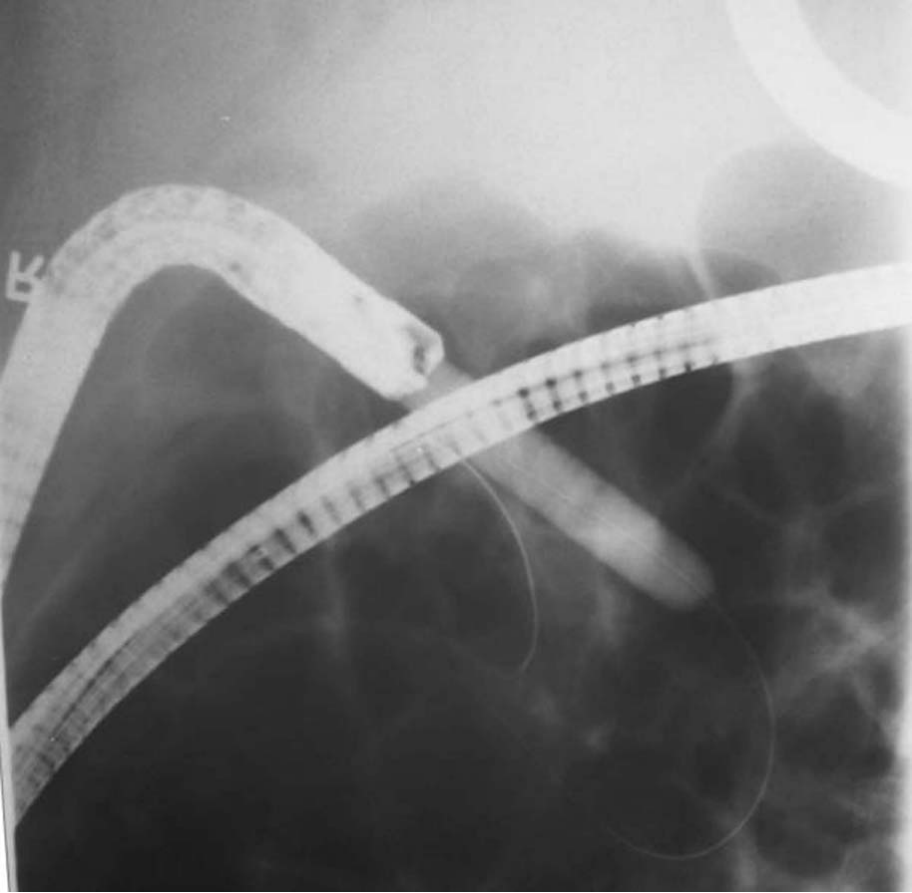
**Passing AS with the help of dilatation balloon**. After filling dilatation balloon at the level of stenosis, it is pulled up to the tip of endoscope. Then, endoscope and balloon are pushed together. Note the endoscope and the balloon are on the same direction.

ERCP was performed with therapeutic duodenoscope (Olympus, TJF 240, Japan) in prone position. The proximal side of stenosis was carefully inspected in all patients with DD/AS. ERCP was carried out when the biliary orifice was detected on proximal side without an effort to pass beyond stricture (Figure 4). The procedure was continued when DD/AS could be passed by using therapeutic duodenoscope. When we were unable to pass stricture by therapeutic duodenoscope, it was replaced with a small caliber diagnostic duodenoscope (Olympus, JF 240, Japan) was. If this maneuver failed, dilatation with 12-18 mm TTS balloon (CRE wire guided esophageal balloon dilator, 240 cm, 7.5 fr, Boston Scientific, Cork, Ireland) was performed and another effort was made to pass beyond stricture. In patients whose strictures could not be passed via this effort, a proton pump inhibitor was initiated twice daily and 10 days later, the procedure was re-attempted with diagnostic duodenoscope. Dilatation was repeated if necessary. In some patients, we were able to pass to the second part of the duodenum via a special maneuver: following balloon dilatation, balloon was pulled back to the tip of endoscope and on the direction guidewire points, endoscope was pushed with balloon to pass stricture (Figure 5). The procedure was tested in both prone an supine positions since it was observed that the procedure gets easier when patient takes supine position.

**Figure 6:**
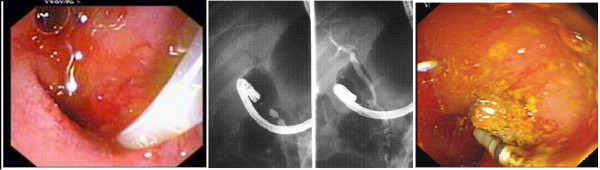
**Cannulation of ectopic CBD opening in case of AS**. Blind cannulation while endoscope is proximal to AS (A). Tight AS and air in the bile ducts (left); cannulation on the stomach side shown (right)(B). Stone removal on the stomach side in the same patient (C).

**Figure 7:**
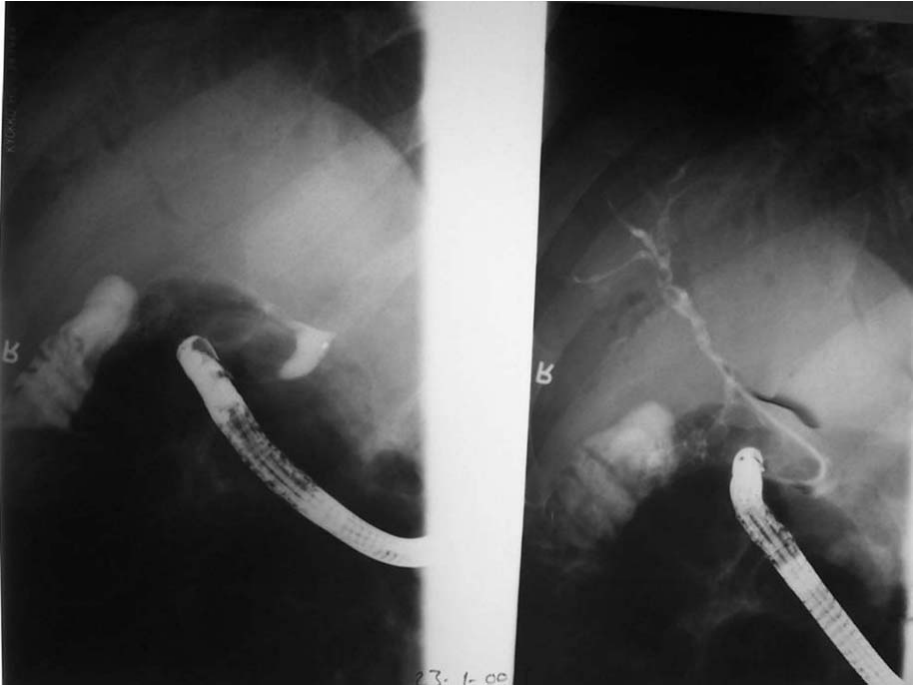
**Cannulation in a patient with gastroenterostomy**. Side-to-side HJ is on view.

Cannulation was attempted in all the cases at the site where the bile duct was likely to open (i.e: just beyond AS)(Figure 6). The procedure was accepted as "failed" when cannulation could not be achieved on two consequtive sessions. Then, examination with diagnostic gastroscope was made to determine whether these patients have opening anomaly. In patients with simple gastroenterostomy, an attempt was first made through pylorus. If apical stricture could not be passed, afferent loop was intubated to continue with the procedure (Figure 7). The patients who had undergone Billroth II gastroenterostomy because of apical stenosis as understood from their postoperative reports were also involved in the study although they did not have apical stenosis at the time. The bile duct orifice was reached through afferent loop in these patients. EO-CBD-DB was diagnosed when the pancreatic and biliary orifices opened separately and a typical hook-shaped cholangiographic view could be obtained.

Therapeutic ERCP was performed via standard techniques in patients with retracted papilla or when papilla is in usual place. In patients with opening anomaly, based on our previous experience, endoscopic sphincterotomy was not attempted since perforation and bleeding are common during this practice. Instead, ERCP was performed following dilatation with 8-15 mm dilatation balloons. In patients whose stone could not be retrieved, a nasobiliary drain (NBD) was placed if extracorporeal schockwave lithotripsy (ESWL) was planned since these stones are usually broken into pieces that soil the bile ducts during lithotripsy. Those patients whose stones were not amenable to extraction were referred to surgery after maintaining the bile flow by NBD or plastic stent.

### Statistics

The Statistical Package for Social Sciences (SPSS v.11.0.0) for Windows was used for statistical analysis. Student's *t *test was performed for parametric values, and Pearson chisquare and Mann-Whitney *U *tests for nonparametric values. Findings are expressed as mean ± standard deviation, and *P *values < 0.05 were accepted as statistically significant. The study was approved by the hospital Ethics Committee and Review Board and a written consent was also obtained from all the patients for the publication of data and figures.

## Results

ERCP was performed in 96 DD/AS patients (79 male, 17 female, mean age 58.5, range: 30-87) (1.32% of total 7276 ERCP procedures) during the study period.

The second part of the duodenum could be reached without dilatation in 62 patients (64.6%). Endoscope was passed to the duodenum after balloon dilatation in 14 patients (14.6%). In 4 of these patients the maneuver described above was carried out to reach the duodenum. ERCP was performed intubating the afferent loop in 5 patients (5.21%) with simple gastroenterostomy (2 patients, one with opening anomaly and the other without) or Billroth II (3 patients, all with opening anomaly). In 9 patients (9.4%), ERCP was achieved by cannulation of the bile duct proximal to AS. An apparent biliary opening could be observed proximal to stenosis in 6 of them, however, ERCP was performed through estimated opening spot proximal to stenosis in 3. The procedure was failed in 6 patients (6.3%) since we were unable to reach the duodenum despite dilatation. There was no complication related to dilatation.

Papilla was not seen at its normal location in the second part of the duodenum, instead, it was found in the bulb in 74 of 96 (77.1%) DD/AS patients (i.e: opening anomaly group. 63 male, 11 female, mean age 56.9, range: 30-87). Notably, pancreas and bile ducts opened separately in all of these subjects. There was no opening anomaly in remaining 22 (22.9%) patients (16 male, 6 female, mean age 63.5, range: 32-84). Half of these patients had normal papilla moved towards the bulb, and the other half had a normally located papilla.

Table 1 shows presentations, surgical histories and diagnoses beside demographic data. In the opening anomaly group, the problem was usually related with the bile duct stone while in the group of no opening anomaly, malignant strictures were more common. Consistent with these findings, jaundice was the most common presentation in the group of no opening anomaly whereas in the anomaly group biliary pain and cholangitis were the main issues.

**Table 1:** Clinical features of the patients.

	Opening anomaly group (n:74)	No opening anomaly group (n:22)	P
Age, mean, range	56.9 (30-87)	63.5 (32-84)	NS
Gender, M/F	63/11	16/6	< 0.05

Presentation, n (%)			< 0.05
Gall stone, biliary problem	38 (51.4)	5 (22.7)	
Operation*, biliay problem	30 (40.5)	6 (27.3)	
Jaundice	4 (5.4)	10 (45.5)	
Chronic pancreatitis, jaundice	2 (2.7)	1 (4.5)	

Surgical history	30 (40.5)	6 (27.3)	NS
No operation	44 (59.5)	16 (72.7)	
Only cholecystectomy	17 (23.0)	3 (13.6)	
Only simple GE	2 (2.7)	3 (13.6)	
Only BII	1 (1.4)		
Cholecystectomy +choledocotomy	1 (1.4)		
Cholecystectomy + simple GE	3 (4.1)		
Cholecystectomy + BII	2 (2.7)		
Cholecystectomy + HJ	3 (4.1)		
Cholecystectomy + simple GE + HJ	1 (1.4)		

Diagnosis			< 0.05
Choledocholithiasis	71 (95.9)	10 (45.5)	
Biliary injury	1 (1,4)	2 (9.1)	
Benign biliary stricture	2 (2,7)	1 (4.5)	
Malignant biliary stricture	0 (0,0)	9 (40.9)	
Pancreatic head carcinoma		4 (18.2)	
Klatskin tumor		3 (13.6)	
Ampullary carcinoma		2 (9.1)	

*Cholecystectomy, biliodigestive surgery and/or gastroenterostomy

GE: gastroenterostomy; BII: Billroth II; HJ: hepaticojejunostomy

Of note, substantial number of patients in both groups had a history of operation due to biliary problems. Thirty (40.5%) patients with DD/AS had undergone a surgery and 27 (36.5%) of them had cholecystectomy. Seventeen out of 27 (23%) had only cholecystectomy while 10 (13.5%) had either simple GE or Billroth II or hepaticojejunostomy additionally. The frequency of biliodigestive surgery was higher in opening anomaly group compared to no anomaly group (Table 1).

The diagnoses on Table 1 were not only made by ERCP but also the other modalities when ERCP failed. ERCP diagnosis is absent for 5 patients in anomaly group and for a single patient in no anomaly group because apical stenosis did not allow to pass beyond or cannulation of the bile duct could not be achieved. Opening anomaly was diagnosed endoscopically in 2 of them. Four patients were found to have opening anomaly and CBD stone by means of MRCP (one had ERCP diagnosis also). PTC demonstrated opening anomaly in a single patient who had bile duct stone shown by transabdominal ultrasound. These 5 patients were treated by surgery. A 32-year-old male who did not have opening anomaly but CBD stone on MRCP was referred to surgery, too.

Thirteen (17.6%) patients in opening anomaly group were given to surgery. Apart from 5 patients in whom apical stenosis could not be passed and bile ducts could not be cannulized, 6 more patients whose CBD stones were not considered amenable to retrieval endoscopically were referred to surgery. Additionally, 2 patients with chronic pancratitis and associated biliary stricture not suitable for endoscopic treatment also underwent operation. Another patient with biliary stricture due to the injury during laparoscopic cholecystectomy was not given to surgery since the level of stricture was high. Treatment of this patient by dilatation and stenting still continues. In 60 (81.1%) of the patients with opening anomaly, treatment was completed after an average of 1.7 (range:1-6) endoscopic procedures. ESWL was used as an additional modality in 4 (6.7%) of them. The mean follow-up period was 24.8 months (range: 2-46) for the patients whose endoscopic treatment was completed. Endoscopic re-treatment was needed in 12 out of 60 (20%) due to recurrent biliary problems.

## Discussion

The results of this study revealed that almost 80% of the patients with DD/AS who were presented with biliary problems have opening anomaly (EO-CBD-DB). Endoscopic treatment is possible in these patients majority of whom have CBD stone-related problems.

Of note, these patients are usually elderly males. In our previous series [9], 49 out of 53 patients were male in accordance with the series of Lee HJ et al. [7] and Lee SS et al. [8] in which 7 of 8, and 15 of 18 patients were males respectively. Case presentations also support this finding.

In a substantial group of patients with opening anomaly, there is a bulbar stenosis occasionally defined as duodenal deformity which in fact can be better described as apical stenosis. The incidence of apical stenosis was 64% (34 out of 53 cases of ectopic papilla in the bulb) in our previous series [9]. The number was small possibly due to retrospective nature of the study. However, when we study the patients with apical stenosis prospectively, the incidence goes up to 100% meaning we never saw a patient with opening anomaly who do not have apical stenosis or deformity. Lee HJ et al. [7] described duodenal deformation in 5 of 8 patients and active duodenal ulcer in a single patient. On the other hand, Lee SS et al. [8] reported 13 patients with active duodenal ulcer 9 of whom had duodenal deformity in a total of 18 cases. These studies are also retrospective case series and the ratio may be low because of incomplete records.

The cause of apical stenosis in patients with opening anomaly is not clearly known. Lee HJ et al. [7] linked the frequent development of dudeonal ulcer and deformity to constant exposure of duodenal bulb to bile acid. On the other hand Lee SS et al. [8] speculated that the bile acids in high pH damage gastric mucosa [14]. They also suggested that bicarbonate in the pancreatic secretion contributes to rising pH in the bulb thus leading to recurrent duodenal ulcer and deformity. Essentially, the presence of pancreatic secretion in addition to bile seems to be necessary for the development of deformity, ulcer and apical stenosis in this part. If the bile alone had such an effect, the similar endoscopic findings would be seen in patients with choledochoduodenostomy. However, it is known that this operation performed for years does not result in ulcer formation.

There is a significant rate of biliodigestive operation history in these patients. The patients who had opening anomaly have a higher rate of gall bladder and biliary operations beside the procedures that ease gastric emptying than those with apical stenosis alone (Table 1). It is conceivable that these patients underwent gastroenterostomy with (Billroth II) or without (simple) gastric resection possibly due to EO-CBD-DB. A cholecystectomy rate of 5/8 and 7/18 was reported by Lee HJ [7] and Lee SS [8], respectively. However, no biliodigestive surgery was reported in their series. This is possibly because these studies are retrospective. Also, the patients with apical deformity and stenosis in whom endoscopic procedures failed or the patients with EO-CBD-DB among those who underwent simple GE or BII GE were not reported in these series.

The presentations and diagnoses of the patients with no opening anomaly are consistent with those of an advanced age group. Malignancies are more common in this age group than the younger group. On the other hand, the primary problem of the patients with opening anomaly is biliary stones and related issues (Table 1). Likewise, biliary stones and cholangitis were reported to be the main presentations in other series [7,8]. The frequency of malignancy does not seem to be increased in these series.

Given the difficulty of endoscopic procedures in the presence of apical stenosis requiring more than one session and nearly 20% recurrence rate, it is arguable that the ideal treatment is endoscopic or not. It may be suggested that correcting apical stenosis, removing biliary stone and performing a biliodigestive intervention all together by surgery would be a better alternative way of treatment. However, considering the patients who had previous HJ, surgical treatment may not overall be a better option. All of our patients who had undergone surgery had side-side HJ.

Choledochoduodenostomy is not an appropriate choice for these patients due to deformity of the bulb. End-to-side HJ is another alternative, however, it is known that this operation on other indications has been complicated by anastomotic stricture in 10-30% of the patients on long-term follow-up [15,16]. Percutaneous treatment has been described in this case although the recurrence is frequent [17,18]. The long-term outcome of the treatment with balloon endoscopy is not known yet [19,20]. If an operation will be performed, our choice is side-to-side HJ not to hinder a future ERCP assistance or if end-to-side HJ is to be performed, then leaving a permanent access loop would be a better approach. In addition, taking likely comorbidities of these elderly patients into account, endoscopic treatment seems to be a safer alternative to surgery.

## Conclusions

ERCPist should consider the possibility of EO-CBD-DB in the presence of DD/AS when he/she could not see the papilla at its normal location in a middle or advanced aged patient with a history of biliodigestive surgery who has air in the bile ducts on imaging studies and presenting with biliary symptoms. Even though apical stenosis occasionally renders the procedure difficult in this patient group, ERCP can be performed safely with balloon dilatation. The comparison of long-term efficacies of endoscopic and surgical treatments merits future studies.

## Competing interests

The authors declare that they have no competing interests.

## Authors' contributions

EP performed the endoscopic procedures, participated in the conception and design of the study. SD performed the endoscopic procedures, participated in the analysis and interpretation of the data. CC participated in the analysis and interpretation of the data and drafted the manuscript. BC performed the endoscopic procedures, participated in the analysis and interpretation of the data. YO participated in the analysis and interpretation of the data.

SK participated in the analysis and interpretation of the data. NS made critical revision of the article for important intellectual content. BS performed the endoscopic procedures and final approval of the article. All authors read and approved the final manuscript.

## Pre-publication history

The pre-publication history for this paper can be accessed here:

http://www.biomedcentral.com/1471-230X/10/142/prepub
